# The glycocalyx: a novel diagnostic and therapeutic target in sepsis

**DOI:** 10.1186/s13054-018-2292-6

**Published:** 2019-01-17

**Authors:** Ryo Uchimido, Eric P. Schmidt, Nathan I. Shapiro

**Affiliations:** 1 Department of Emergency Medicine, Beth Israel Deaconess Medical Center, 1 Deaconess Road, Boston, MA 02215 USA; 2 Pulmonary Sciences and Critical Care Medicine, University of Colorado School of Medicine, 13001 E 17th Pl, Aurora, CO 80045 USA

**Keywords:** Glycocalyx, Vascular endothelial cell, Glycocalyx degradation, Heparan sulfate, Syndecan, Hyaluronan, Fluid therapy, Fibroblast growth factor, Sepsis

## Abstract

The glycocalyx is a gel-like layer covering the luminal surface of vascular endothelial cells. It is comprised of membrane-attached proteoglycans, glycosaminoglycan chains, glycoproteins, and adherent plasma proteins. The glycocalyx maintains homeostasis of the vasculature, including controlling vascular permeability and microvascular tone, preventing microvascular thrombosis, and regulating leukocyte adhesion.

During sepsis, the glycocalyx is degraded via inflammatory mechanisms such as metalloproteinases, heparanase, and hyaluronidase. These sheddases are activated by reactive oxygen species and pro-inflammatory cytokines such as tumor necrosis factor alpha and interleukin-1beta. Inflammation-mediated glycocalyx degradation leads to vascular hyper-permeability, unregulated vasodilation, microvessel thrombosis, and augmented leukocyte adhesion. Clinical studies have demonstrated the correlation between blood levels of glycocalyx components with organ dysfunction, severity, and mortality in sepsis.

Fluid resuscitation therapy is an essential part of sepsis treatment, but overaggressive fluid therapy practices (leading to hypervolemia) may augment glycocalyx degradation. Conversely, fresh frozen plasma and albumin administration may attenuate glycocalyx degradation. The beneficial and harmful effects of fluid and plasma infusion on glycocalyx integrity in sepsis are not well understood; future studies are warranted.

In this review, we first analyze the underlying mechanisms of glycocalyx degradation in sepsis. Second, we demonstrate how the blood and urine levels of glycocalyx components are associated with patient outcomes. Third, we show beneficial and harmful effects of fluid therapy on the glycocalyx status during sepsis. Finally, we address the concept of glycocalyx degradation as a therapeutic target.

## Introduction

The glycocalyx is a gel-like layer lining the luminal surface of endothelial cells, composed of membrane-bound proteoglycans, glycoproteins, glycosaminoglycans, and adherent plasma proteins [1]. It performs several functions necessary for vascular homeostasis: it regulates vascular permeability and microvascular tone, inhibits microvascular thrombosis, and helps regulate leukocyte adhesion on the endothelium [2–4]. During sepsis, glycocalyx degradation occurs due to a combination of pathophysiologic insults, potentially compounded by iatrogenic effects of accompanying fluid resuscitation practices [5–9]. Glycocalyx fragments shed into the blood during sepsis may serve as clinically relevant biomarkers, given the pathophysiologic implications of glycocalyx degradation. The degradation of glycocalyx is also thought to contribute to microcirculatory dysfunction in sepsis [10]. The objectives of this article are to review: 1) human studies that investigate the association of biomarkers of glycocalyx degradation with clinical outcomes, 2) mechanisms of glycocalyx degradation, and 3) the potential of the glycocalyx as a therapeutic target in sepsis.

## Background

### Components and structure

**Fig. 1:**
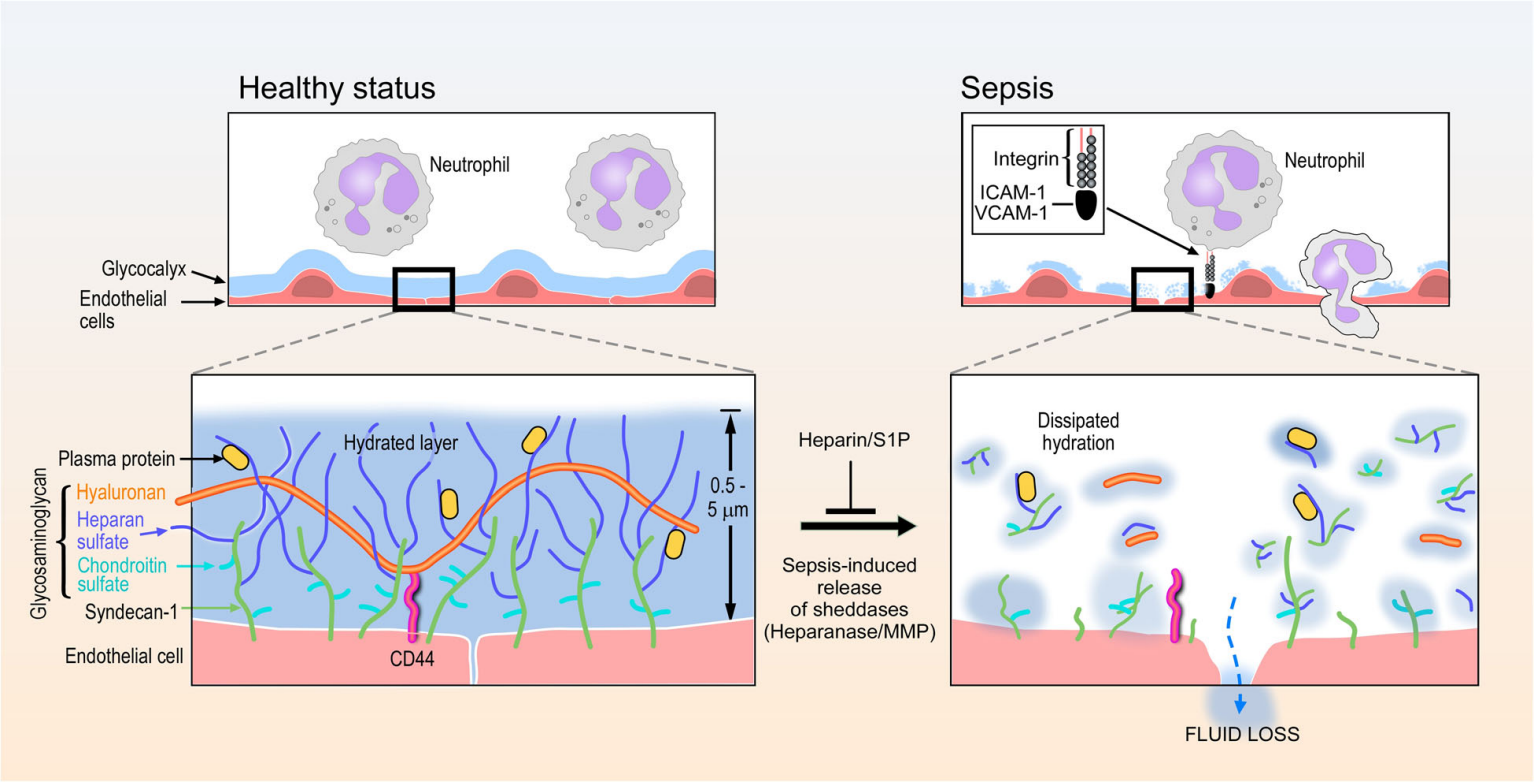
Endothelial glycocalyx structure during health and degradation during sepsis. *MMP* metalloproteinase, *S1P* sphingosine-1-phosphate, *ICAM-1* intercellular adhesion molecule 1, *VCAM-1* vascular cell adhesion molecule 1

The glycocalyx is a layer that lines the luminal surface of vascular endothelial cells (Fig. 1). The thickness and structure of the glycocalyx vary across different species, vascular beds, organs, and blood flow rates [11]. Its observed thickness in humans is approximately 0.5 to 5.0 μm [12–18]. The glycocalyx consists of proteoglycans (PG), glycoproteins bound with sialic acid, glycosaminoglycans (GAG), and associated plasma proteins [1]. Proteoglycans are core proteins anchored to the apical membrane of endothelial cells, to which several GAG chains are covalently attached [19]. There are many different types of proteoglycans, but syndecan-1 (a subtype of syndecan family from 1 to 4) is a primary target of prior endothelial glycocalyx investigations [20]. GAG chains that bind to proteoglycans include heparan sulfate (a major component of GAGs, comprising over 50% of GAGs in glycocalyx), chondroitin sulfates, dermatan sulfates, and possibly keratin sulfates [21]. These sulfated GAGs are negatively charged, enabling electrostatic interaction with plasma proteins [17]. In contrast, hyaluronan (hyaluronic acid) is a large linear molecule that does not bind to proteoglycans, but instead interacts with cell-membrane CD44. Hyaluronan also differs from other GAGs as it is not sulfated and is therefore uncharged. However, hyaluronan can form complexes with other sulfated GAGs, enabling it to sequester water and stabilize the gel-like structure of the glycocalyx [21]. Proteins such as albumin, fibrinogen, fibronectin, thrombomodulin, antithrombin III, superoxide dismutase, and cell-adhesion molecules all interact with GAGs.

### Glycocalyx function in health and disease

In the healthy state, the glycocalyx serves as a barrier opposing vascular permeability, in part by serving as a negatively charged molecular sieve [3]. This “meshwork” limits transvascular movement of negatively charged and/or larger than 70-kDa molecules. By establishing a transvascular albumin gradient, the intact glycocalyx regulates transvascular fluid flux (in accordance with the so-called “revised” Starling equation) [3, 4, 22]. Additionally, the glycocalyx senses fluid shear forces and transmits these forces to endothelial cells, initiating nitric oxide-mediated vasorelaxation. The glycocalyx provides anti-coagulant and anti-adhesive effects on the surface of endothelial cells. Moreover, it can shield endothelial cells from oxidative stress. In sepsis, the glycocalyx is degraded and cannot perform its normal functions, which leads to enhanced vascular permeability, tissue edema, augmented leukocyte adhesion, platelet aggregation, and dysregulated vasodilation (Fig. 1) [20]. Thus, it is hypothesized that these dysfunctions of glycocalyx, mainly resulting from its degradation, have a role in the early diagnosis and prognosis of sepsis; and restoration of the glycocalyx is a potential therapeutic target.

### Degradation of the endothelial glycocalyx during sepsis

In sepsis, the degraded glycocalyx layer becomes thinner and more sparse, allowing plasma proteins (e.g., albumin) and fluid to move across the vascular wall, leading to tissue edema formation (Fig. 1) [6, 23]. This degradation releases glycocalyx components (such as syndecan-1, heparan sulfate, hyaluronan, chondroitin sulfates) into the plasma. Several enzymes mediate this degradation. Heparanase directly cleaves the heparan sulfate chains attached to core proteoglycans. Metalloproteinases (MMPs) are known to cleave proteoglycans (e.g. syndecan-1) directly from the endothelial cell membrane [18, 24]. These specific enzymes are activated in inflammatory states by reactive oxygen species (ROS) and pro-inflammatory cytokines such as tumor necrosis factor alpha (TNF-α) and interleukin-1beta (IL-1β) [18, 20, 24–26]. Elevated heparanase expression can secondarily increase MMP expression in myeloma cells [27], suggesting cross-talk between sheddases.

Many preclinical and clinical studies have demonstrated a decrease in the thickness of the glycocalyx in sepsis. For example, Wiesinger et al. [28] found that mice treated with intravenous lipopolysaccharide (LPS) had a significant reduction in aorta glycocalyx thickness compared to controls (0.27 ± 0.012 μm vs 0.14 ± 0.017 μm, *p* < 0.0001). Furthermore, human umbilical venous endothelial cells showed a 50% reduction in the glycocalyx thickness when the cells were treated with TNF-α or LPS in vitro. Nieuwdorp et al. [29] used a human volunteer endotoxin model to study the glycocalyx in sepsis. In 13 healthy human subjects who received low-dose intravenous endotoxin, a significant decrease in sublingual glycocalyx thickness was observed (0.60 ± 0.1 μm to 0.30 ± 0.1 μm, *p* < 0.01) with a concurrent elevation of plasma hyaluronan (62 ± 18 to 85 ± 24 ng/mL, *p* < 0.05).

### Vascular heterogeneity of the glycocalyx layer

Endothelial cells from different vascular beds (organ-specific) and vascular locations (arteriole, capillary, and venule) display different glycocalyxes [30]. In vivo, pulmonary artery endothelial cells are enriched with α-galactose carbohydrates, while pulmonary microvascular endothelial cells are enriched with α- and β-N-acetylgalactosamine carbohydrates [31]. Schmidt et al. [18] found that the thickness of pulmonary microvascular endothelial glycocalyx of healthy mice (*n* = 43, 1.67 ± 0.09 μm) is substantially greater than that of cremasteric microvessels (*n* = 9, 0.67 ± 0.08 μm). Furthermore, mechanisms of glycocalyx shedding may differ across vascular beds, as septic induction of pulmonary endothelial heparanase was not observed in cremasteric endothelial cells [18]. The geographic heterogeneity of glycocalyx structure and degradation is a focus of current investigations.

## Measures of glycocalyx degradation in sepsis

### Direct bedside imaging of glycocalyx degradation during sepsis

Orthogonal phase spectrometry (OPS) or sidestream dark field (SDF) imaging are intravital microscopy imaging techniques capable of assessing sublingual microvascular thickness at the bedside [16, 32]. The perfusion boundary region (PBR), a SDF-measured parameter that is inversely related to the glycocalyx thickness, has been proposed as a proxy of the glycocalyx thickness. Donati et al. [33] reported that the discriminative performance of PBR for the presence of sepsis as the area under the receiver operating characteristic curve (AUC) is 0.67 (95% CI 0.52–0.82, *p* = 0.05). While immediate assessment of glycocalyx degradation via handheld microscopy may be a promising diagnostic/prognostic test in early sepsis, concerns remain for inter-observer reliability and relevance of the sublingual microcirculation as a marker of clinically relevant organ injury. As such, further studies are required.

### Quantification of septic glycocalyx degradation using circulating biomarkers

#### Syndecan-1

Studies demonstrate that syndecan-1 shedding is associated with both sepsis presence and severity (Table 1). Nelson et al. [34] reported that septic shock patients admitted to the intensive care unit (ICU; *n* = 18) had a significantly higher median level of syndecan-1 compared to healthy controls (n = 18; 246 [interquartile range (IQR) 180–496] ng/mL vs 26 [IQR 23–31] ng/mL, *p* < 0.001). They also detected a correlation between syndecan-1 level with Sequential Organ Failure Assessment (SOFA) score (*r* = 0.48, *p* < 0.05) and Cardiovascular SOFA score (*r* = 0.69, *p* < 0.01) during the first 24 h of admission. Despite these findings, there was no association between the median level of syndecan-1 and mortality. Steppan et al. [35] compared syndecan-1 levels among three groups; healthy volunteers (*n* = 18), patients after major abdominal surgery (*n* = 28), and severe sepsis/septic shock patients (*n* = 104). The mean syndecan-1 levels for both the surgery group (50.5 ± 46.9 ng/mL) and sepsis group (160 ± 109 ng/mL) were higher compared to controls (20.5 ± 5.05 ng/mL) (*p* = 0.01 and *p* < 0.001, respectively). Sallisalmi et al. [36] found that ventilated adult patients with septic shock (*n* = 20) in the ICU had significantly higher median syndecan-1 than healthy controls. They also reported a significant correlation of syndecan-1 levels with SOFA score on day 1 of ICU admission (*r* = 0.654, *p* < 0.002). Ostrowski et al. [37] showed a correlation between syndecan-1 levels and SOFA scores across a range of sepsis severities: local infection (*r* = 0.40, *p* = 0.004), sepsis (*r* = 0.34, *p* = 0.002), severe sepsis (*r* = 0.28, *p* = 0.009), and septic shock (*r* = 0.60, *p* = 0.051).

**Table 1:** Clinical studies investigating the association of glycocalyx levels with clinical outcomes

	Year	Authors	Design	Patients population	Patient classification	N	Outcome	Brief result
Syndecan-1	2008	Nelson et al. [34]	Obsevational	Intensive care unit	Cont vs Septic shock	18 vs 18	septic shock	Septic shock significantly higher median syndecan-1 vs healthy controls (246 [IQR 180-496] vs 26 ng/ml, [23 - 31], p<0.01)
					Septic shock	18	Mortality	No significant association of syndecan-1 with mortality
					Septic shock	18	Severity	Septic shock significantly higher median syndecan-1 vs healthy controls (246 ng/mL, [180-496] vs 26 ng/ml, [23 - 31], p<0.01)
	2011	Steppan et al. [35]	Obsevational	(not specifically referred )	Cont vs Abdominal surgery vs Severe sepsis or septic shock	18 vs 28 vs 104	Severe sepsis or septic shock	Septic patients significantly higher mean syndecan-1 than healthy control (160±109 vs 20.5± 5.1 ng/mL, p<0.001) and than surgery patients (50.5±46.9 ng/mL, p=0.001)
					Severe sepsis or septic shock	104	Level of IL-6	Syndecan-1 significantly correlated with IL-6 (p=0.004)
	2012	Sallisalmi et al. [36]	Obsevational	Intensive care unit	Cont vs Septic shock	20 vs 20	septic shock	Septic shock higher median syndecan-1 vs controls (p<0.0001)
					Septic shock	20	Severity	syndecan-1 corelates with SOFA score (r=0.654, p<0.002)
					Septic shock	20	Level of VAP-1	syndecan-1 correlated with VAP-1 level (r=0.729, p<0.0001)
	2013	Ostrowski et al. [73]	Obsevational	Intensive care unit	Experimental endotoxemia vs Severe sepsis or septic shock	9 vs 20	Presence of Severe sepsis or septic shock	Septic patients had higher syndecan-1 vs experimental endotoxemia patients (172 ± 102 VS 51 ± 12 ng/mL, p <0.05)
					Severe sepsis or septic shock	20	Severity	No significant correlations of syndecan-1 level with SOFA score (r=0.42, p=NS) and SAPS2(r=0.04, p=NS)
					Severe sepsis or septic shock	20	Level of biomarkers	Syndecan-1 correalted with noradrenaline (r=0.45, p=0.045), adrenaline(r=0.62, p=0.004), lactate(r=0.77, p<0.001), APTT(r=0.63, p=0.005), INR(r=0.57, p=0.013)
	2014	Donati et al. [74]	RCT	Intensive care unit	Non-leukodepleted RBC transfusion vs leukodepleted RBC transfusion	10 vs 10	Level of syndecan-1	In non-leukodepleted RBC transfusion group, the median level of syndecan-1 was significantly increased. No significant association between syndecan-1 and leukodepleted RBC transfusion
	2014	Johansson et al. [75]	Obsevational	Intensive care unit	Severe sepsis or septic shock	67	Treatment with noradrenalin infusion at the time of blood sampling	No significant association between syndecan and noradrenaline infusion. (p=0.902, data not shown)
					Severe sepsis or septic shock not treated with noradrenaline infusion at blood sampling	53	Level of biomarkers	Significant correlations of syndecan-1 level with noradrenaline (r=0.29, p=0.034), sTM(r=0.35, p=0.01), hcDNA(r=0.29, p=0.038), protein C(r=-0.56, p<0.0001), tPA(r=0.44, p=0.001), sVEGFR1(r=0.56, p<0.0001), Ang-1(r=-0.51, p<0.001), Ang-2(r=- 0.40, p=0.003), TFPI(r=0.44, p=0.001), platelet count(r=-0.45, p=0.001), creatinine(r=0.34, p=0.012), bilirubin(r=0.45, p=0.001)
					Severe sepsis or septic shock not treated with noradrenaline infusion at blood sampling	53	Presence of septic shock	Significant correlation of syndecan-1 level with shock. (r=0.40, p=0.003)
					Severe sepsis or septic shock not treated with noradrenaline infusion at blood sampling	53	Severity	There were significant correlations of syndecan-1 level with SOFA score (r=0.33, p=0.027) and SAPS2 (r=0.33, p=0.015)
	2015	Straat et al. [67]	Obsevational	Intensive care unit	non-bleeding critically ill patients	33	Level of syndecan-1	The median level of syndecan-1 after fresh frozen plasma transfusion was significantly lower than the level before the transfusion (565 [IQR 127–1176] ng/mL vs 675 [IQR 132–1690] ng/mL, p=0.01)
	2015	Ostrowski et al. [76]	Obsevational	Intensive care unit	Severe sepsis or septic shock	184	Coagulopathy	There were siginificant associations of syndecan-1 with TEG R-time (β: 0.64 ± 0.25, p=0.013), TEG MA (β: -1.78 ± 0.87, p=0.042) and FF MA (β: -0.84 ± 0.42, p=0.045)
	2015	Ostrowski et al. [37]	Obsevational	Department of internal medicine	No infection vs Local infection vs Sepsis vs Severe sepsis vs Septic shock	50 vs 63 vs 95 vs 100 vs 13	Presence of severe sepsis or septic shock	Septic shock (61 ng/mL, [IQR 39 - 119], p<0.05), severe sepsis (61 ng/mL, [IQR 35 - 95], p<0.05) had a significantly higher median level of syndecan than sepsis (31 ng/mL, [IQR 22 - 50])
					No infection or Local infection or Sepsis or Severe sepsis or Septic shock	321	28 days mortality	There was a significant association of the median syndecan-1 level with comulative survival over 28days. (p=0.029)
	2016	Anand et al. [77]	Obsevational	Intensive care unit	Cont vs Sepsis vs Severe sepsis vs Septic shock	50 vs 15 vs 45 vs 90	Presence of sepsis	Septic shock (653.5 ng/mL, [IQR 338.93 - 1430.23], p<0.001), severe sepsis(342.1 ng/mL, [IQR 130 - 568.1], p<0.001) and sepsis patitents(85.78 ng/mL, [IQR 40.16 - 141.2], p<0.001) had a significantly higher median level of syndecan than healthy control(28.15 ng/mL, [IQR 7.47 - 45.7])
					Sepsis or Severe sepsis or Septic shock	150	90 days mortality	Non-survivor had a significantly higher median level of syndecan-1 than survivor (782 ng/mL, [IQR 235.5 - 1514.31 vs 412.3 ng/mL, [IQR 135.25 - 855.34]; p=0.007). AUC of syndecan-1 level for mortality was 0.644 [95%CI 0.54-0.74]. There was a significant association of the cut off level of syndecan-1:625 ng/ml on DAY 1 ICU admission with comulative survival on Kaplan-Meier plot (p=0.003)
					Sepsis or Severe sepsis or Septic shock	150	Severity	There were significant correlations between syndecan-1 levels level with SOFA score (r=0.437, p<0.001) and APACHE2 score (r=0.294, p<0.001).
	2016	Puskarich et al. [38]	Obsevational	Emergency department	Severe sepsis or septic shock	175	Intubation	Intubated patients had syndecan-1 similar to non-intubated patients (181 ng/mL [61 - 568] vs 141 ng/mL [46 - 275], p=0.06)
					Severe sepsis or septic shock	175	Mortality	Non-survivor had a significantly higher level of syndecan-1 than survivor ( 223 ng/mL [67 - 464] vs 142 ng/mL [38 - 294], p=0.04)
					Severe sepsis or septic shock	175	Development of AKI	Patients with AKI developlment had a significantly higher level of syndecan-1 than those without AKI development (193 ng/mL [IQR, 63 - 441] vs 93 ng/mL [IQR 23 - 187], p<0 .001)
Heparan sulfate	2011	Steppan et al. [35]	Obsevational	not shown	Cont vs Abdominal surgery vs Severe sepsis or septic shock	18 vs 28 vs 104	Severe sepsis or septic shock	Septic patients had a significantly higher mean level of heparan sulfate than healthy control (3.23 ± 2.43 μg/ml vs 1.96 ± 1.21 μg/ml, p = 0.03). Septic patients had a significantly lower mean level of heparan sulfate than surgery patients (3.23 ± 2.43 μg/ml vs 7.96 ± 3.26 μg/ml, p <0.001)
	2014	Nelson et al. [39]	Obsevational	Intensive care unit	Cont vs Septic shock	24 vs 24	septic shock	Septic shock patients had a significantly higher mean level of heparan sulfate than control (p<0.001, data not shown)
					Septic shock	24	Severity	There was a significant correlation between heparan sulfate level with SOFA score (r=0.47, p=0.02)
					Septic shock	24	Mortality	Non-survivor had a significantly higher mean level of heparan sulfate than survivor (p=0.02, data not shown)
					Septic shock	24	Level of biomarkers	There were no significant correlations of heparan sulfate level with the levels of IL-6 (r=0.40, p=0.06), IL-10 (r=0.34, p=0.10), CRP (r=-0.19, p>0.3), and MPO (r=0.21, p>0.3)
	2016	Schmidt et al. [41]	Obsevational	Intensive care unit	Severe trauma vs Septic shock	25 vs 30	Septic shock	Septic shock patients had a significantly highe mean level of heparan sulfate than sever trauma patitents (p<0.05, data not shown)
					No AKI development vs AKI development in Septic shock	16 vs 14	Development of AKI	Patient who developed AKI had a significantly higher mean level of heparan sulfate than those who do not (p<0.05, data not shown) Non adjusted AUC for the development of AKI was 0.7634 (p=0.014). Aadjusted AUC was not significant (data not shown)
					Septic shock	30	Mortality	Non-survivor had a significantly higher mean level of heparan sulfate than survivor (p<0.05, data not shown)
								Non adjusted AUC for the mortality was 0.86 (p=0.0009) Adjusted AUC was 0.91 (p=0.0003)
Hyaluronan	2012	Yagmur et al. [42]	Obsevational	Intensive care unit	No SIRS vs SIRS vs Sepsis(sepsis, severe sepsis or septic shock) without cirrhosis	20 vs 33 vs 97	Sepsis	Septic patients(344 μg/ml [IQR 0 – 2641]) had a significantly higher median level of hyaluronan than No SIRS (115.5 μg/L [IQR 10 – 2457], p=0.014) and SIRS patients (168 μg/L [IQR 0 – 2117], p=0.015)
							Severity	Hyaluronan correalted with SOFA score (r=0.278, p=0.001)
							Level of biomarkers	Hyaluronan correlated with Procalcitonin(r=0.46, p<0.001), CRP (r=0.34, p<0.001), IL-6 (r=0.34, p=0.004) and IL-10 (r=0.38, p=0.001)
	2014	Nelson et al. [39]	Obsevational	Intensive care unit	Cont vs Septic shock	24 vs 24	Septic shock	Septic had a significantly higherdisaccharides from hyaluronan than control (p<0.001)
					Septic shock	24	Severity	Hyaluronan correlated with SOFA score (r=0.47, p=0.02)
					Septic shock	24	Mortality	Non survivor had a significantly higher disaccharides from hyaluronan than survivor (p=0.006, data not shown)
	2016	Schmidt et al. [41]	Obsevational	Intensive care unit	Sever trauma vs Septic shock	25 vs 30	Septic shock	Septic shock patients had a significantly higher level of hyaluronan than sever trauma patitents (p<0.05, data not shown)
					No AKI development vs AKI development in Septic shock patients	16 vs 14	Development of AKI	Patient who developed AKI had a significantly higher level of hyaluronan than not (p<0.05, data not shown)
					Septic shock	30	Mortality	Non adjusted AUC for the development of AKI was 0.75 (p=0.018)
								Adjusted AUC was 0.77 (p=0.01)
								Non survivor had a significantly higher level of hyaluronan than survivor (p<0.05, data not shown)
								Non adjusted AUC for the mortality was 0.86 (p=0.0009). Adjusted AUC was not significant (data not shown)

*Abbreviation: SOFA* sequential organ failure assessment, *IL-6* Interleukin-6, *VAP-1* vascular adhesion protein-1, *NS* not significant, *SAPS* simplified acute physiology score, *RCT* randomized control trial, *RBC* red blood cell, *sVEGFR1* soluble vascular endothelial growth factor receptor, *hcDNA* histone-complexed, *sTM* soluble thrombomodulin, *tPA* tissue-type plasminogen activator, *Ang-1* angiopoietin-1, *Ang-2* angiopoietin-2, *TFPI* tissue factor pathway inhibitor, *IQR* interquartile range, *TEG* thrombelastography, *MA* maximum amplitude, *FF* functional fibrinogen, *BUN* blood urea nitrogen, *ICU* intensive care unit, *APACHE* acute physiology and chronic healthevaluation, *AKI* acute kidney injury, *CRP* C-reactive protein, *MPO* myeloperoxidase, *IL-10* Interleukin-10, *AUC* area under the receiver operating characteristic curve

Puskarich et al. [38] compared median syndecan-1 levels at enrollment between patients who required intubation and those who did not and found that syndecan-1 levels in intubated patients were not significantly higher than in non-intubated patients (181 ng/mL [IQR 61–568] vs 141 ng/mL [IQR 46–275], *p* = 0.06). The receiver operating characteristic (ROC) curve analysis showed syndecan-1 levels alone poorly predicted intubation (AUC 0.58, 95% CI 0.48–0.68). However, the syndecan-1 levels in patients who developed acute kidney injury (AKI) were higher than in those who did not (193 ng/mL [IQR 63–441] vs 93 ng/mL [IQR 23–187], *p* < 0.001).

#### Heparan sulfate

Heparan sulfate is also reported as elevated in sepsis. Steppan et al. [35] compared heparan sulfate levels among healthy volunteers (*n* = 18), patients after major abdominal surgery (*n* = 28), and severe sepsis/septic shock patients (*n* = 104). Mean heparan sulfate levels were significantly higher in the surgery group (7.96 ± 3.26 μg/ml, *p* < 0.001) and sepsis group (3.23 ± 2.43 μg/ml, *p* = 0.03) compared to control values (1.96 ± 1.21 μg/ml). Additionally, septic patients had significantly lower mean levels of heparan sulfate than surgery patients (3.23 ± 2.43 μg/ml vs 7.96 ± 3.26 μg/ml, *p* < 0.001). Nelson et al. [39] found fourfold significantly higher levels of heparan sulfate in 24 patients with septic shock compared to 24 controls scheduled for neurosurgery. Additionally, patients who died within 90 days had fivefold significantly higher levels of heparan sulfate compared to survivors. Schmidt et al. [40] investigated plasma levels of heparan sulfate in ICU patients mechanically ventilated with respiratory failure due to altered mental status (*n* = 4), indirect lung injury (*n* = 6), and direct lung injury (*n* = 7). They measured heparan sulfate levels in plasma collected upon ICU admission and found that patients with indirect lung injury had 23-fold higher median levels of heparan sulfate compared to normal donors. These patients also had increased heparan sulfate degradation activity in this (and a prior study [18]), indicating that mechanically ventilated patients with indirect lung injury might have elevated systematic heparanase activity. They also demonstrated that heparan sulfate plasma levels of the 17 ICU patients with mechanical ventilation were positively and significantly correlated with ICU length of stay (*r* = 0.58, *p* = 0.01).

Similarly, Schmidt et al. [41] assessed urine heparin sulfate, which may primarily reflect renal glycocalyx degradation. They compared 30 medical ICU septic shock patients with 25 severe trauma patients in surgical ICU as controls and found significantly higher heparan sulfate levels in the urine of sepsis patients. They also found urine heparan sulfate levels to be highly prognostic for mortality with an AUC of 0.86, which remained significant after adjusting for Acute Physiology and Chronic Health Evaluation (APACHE) II scores (AUC 0.91, *p* = 0.0003). In this study, heparan sulfate was the only GAG that had a significant AUC with mortality after the adjustment; hyaluronic acid and chondroitin sulfate were not prognostic.

#### Hyaluronan

In the study by Schmidt et al. described above [41], the mean hyaluronan concentration in urine in septic shock patients was significantly higher than that from severe trauma patients. Sepsis non-survivors had a significantly higher mean level of hyaluronan at study entry compared to survivors. The ROC analysis showed a strong unadjusted AUC for hospital mortality of 0.86 (*p* < 0.001). Urinary hyaluronan also strongly predicted the later onset of renal failure (AUC 0.75, *p* < 0.02). In a study of plasma hyaluronan, Yagmur et al. [42] classified 150 ICU patients without cirrhosis into three categories; no systemic inflammatory response syndrome (no-SIRS; *n* = 20), SIRS (*n* = 33), and sepsis (*n* = 97). Sepsis patients had a higher median level of hyaluronan (344 ng/mg [IQR 0–2641]) versus no-SIRS (116 ng/mg [IQR 10–2457], *p* = 0.014.) and SIRS (168 ng/mg [IQR 0–2117], *p* = 0.015). However, they did not find a significant association between hyaluronan level and mortality.

## Mechanisms of glycocalyx degradation in sepsis

### Inflammation-induced glycocalyx degradation

Many preclinical and clinical studies have demonstrated an association between inflammatory cytokines such as TNF-α, IL-1β, IL-6, and IL-10 and glycocalyx degradation biomarkers [28, 29, 39, 42–45]. Schmidt et al. [18] showed TNF-α was sufficient to induce glycocalyx degradation in septic mice. Furthermore, by using TNF-α receptor 1-deficient mice, they demonstrated that TNF-α signaling was necessary for glycocalyx degradation. This degradation was mediated by heparanase activation, as TNF-α treatment of lung microvascular endothelial cells increased both heparanase post-translational activation as well as heparan sulfate degradation activity.

Yang et al. [46] reported MMP15 (ADAM15) as a sheddase for CD44 ectodomain both in vivo and vitro. They found that in MMP15 knockdown endothelial cells, the shedding of CD44 induced by LPS treatment was greatly reduced. Similarly, in MMP15 knockout mice, the shedding of CD44 induced by cecal ligation puncture procedure is attenuated. However, the pathway of how MMP15 is activated during sepsis is still unclear in this study.

Recently angiopoietin 2 (Ang-2) has been investigated as a key mediator of glycocalyx degradation. Ang-2 is a protein secreted by endothelial cells in response to inflammatory stimuli. It serves as an intrinsic antagonist of angiopoietin 1 (Ang-1), preventing anti-inflammatory signaling usually induced by Ang-1 activation of its receptor TIE2. Lukasz et al. [47] found that Ang-2 treatment causes a rapid degradation of endothelial glycocalyx both in vivo and vitro, using a human umbilical vein endothelial cell line and mice. Han et al. [30] revealed that inhibiting Ang-2 leads to the reduced shedding of the endothelial glycocalyx and improved survival among mice with sepsis, concluding that the inhibiting Ang-2 and activating TIE2 might be a potential therapeutic target in sepsis.

While inflammatory stimuli can initiate glycocalyx degradation, glycocalyx integrity can also feed-back on the processes of inflammation themselves. Heparan sulfates and syndecan-1 bind to chemokines on the cell surface; release of these chemokines during degradation might amplify inflammation by promoting additional neutrophil recruitment [48–51]. This process has been elucidated in epithelial injury by Li et al. [52], who found that syndecan-1 binds to KC, a CXC chemokine, on the surface of lung epithelial cells. Matrilysin-mediated shedding of syndecan-1 ectodomain bound to KC created transepithelial chemokine gradients and controlled the neutrophil migration to the injured lung tissue. In endothelial cells, the exact balance between the effects of localizing chemokines on the cell surface and the effect of the release of the chemokine after the shedding remains to be further studied [48].

### Impact of fluid resuscitation on septic glycocalyx degradation

Fluid resuscitation is an essential therapeutic treatment for sepsis and septic shock [53]. However, the type of fluid resuscitation used as well as the volume of resuscitation may significantly impact glycocalyx integrity.

#### Data suggest that excessive fluid resuscitation might cause glycocalyx degradation

Hypervolemia has been associated with increased glycocalyx degradation in sepsis. Several preclinical and clinical studies suggest that hypervolemia induces the release of atrial natriuretic peptide (ANP) by the cardiac atrium in response to mechanical wall stress, which in turn may degrade the glycocalyx [54]. Chappell et al. [55] conducted a pilot study using elective cardiac surgery patients with good cardiopulmonary function. They compared serum levels of ANP and biomarkers of glycocalyx degradation (syndecan-1, heparan sulfate, and hyaluronan) before and after volume loading with 6% hydroxyethyl starch 130/0.4 (20 ml/kg; *n* = 9) and acute normovolemic hemodilution (n = 9), in which the amount of blood drawn was simultaneously replaced with similar amounts of 6% hydroxyethyl starch (HES) 130/0.4. The mean ANP level was significantly increased in volume loading group (13.6 ± 6.2 ng/g to 25.1 ± 11.4 ng/g albumin, *p* < 0.05; data normalized to grams per deciliter of plasma albumin) whereas the normovolemic group had no significant increase in the mean ANP level (13.4 ± 3.5 ng/g to 14.8 ± 5.6 ng/g). The volume loading had a corresponding significant increase in mean levels of syndecan-1 (24.8 ± 8.1 to 45.4 ± 14.9 μg/g albumin) and hyaluronan (32.6 ± 5.5 to 56.4 ± 12.2 μg/g albumin) (*p* < 0.05 for each) while the normovolemic group produced no significant increased mean levels of all three biomarkers.

These findings were corroborated in a preclinical study that demonstrated that ANP independently induced glycocalyx degradation [56]. Isolated guinea pig hearts were first infused with Krebs-Henseleit buffer for an equilibration period. Then 6% HES solution was infused into the coronary vascular system for 20 min without ANP (control group, *n* = 6) and with ANP (ANP group, *n* = 6). The ANP group had a 9- to18-fold higher level of syndecan-1 in their coronary effluent at 6, 10, 15, and 20 mins after the start of the HES infusion, compared to the control group. This corresponded to a decrease in glycocalyx thickness in coronary vessels of the isolated guinea pig hearts as observed by electron microscopy. Puskarich et al. [38] investigated the association between syndecan-1 levels of patients with severe sepsis or septic shock and the volume of fluid administered to emergency department patients. They categorized their 175 patients analyzed based on syndecan-1 level into a high group (syndecan-1 level ≥ 240 ng/mL) and low group (syndecan-1 level < 240 ng/mL) and found no difference in total crystalloid volume of fluid administered between high and low syndecan-1 groups (4.0 L [IQR 3.3–5.3] vs 3.5 L [IQR 2.4–5.0], *p* = 0.36). The association between hypervolemia states with glycocalyx degradation in patients with sepsis remains unclear.

While intriguing, a causal association between ANP and glycocalyx shedding remains unproven. For example, Hahn [57] suggested that volume loading only modestly increases plasma concentrations of ANP. In addition, to our knowledge there is no proven mechanism by which ANP causes glycocalyx shedding. Further studies are therefore required.

#### Use of colloids to protect glycocalyx integrity

Albumin, a colloid commonly used for volume resuscitation, has been proposed to be glycocalyx-protective as it carries erythrocyte-derived sphingosine-1-phosphate (S1P) to the endothelium, where it can mediate glycocalyx recovery by suppressing MMP activity [58, 59]. Jacob et al. [60] showed albumin prevents glycocalyx degradation more effectively than HES and 0.9% normal saline in their animal heart model. In this study, they did not actually assess the glycocalyx, but evaluated the effect of albumin and HES on vascular filtration, which reflects glycocalyx degradation. They used isolated guinea pig hearts pretreated with heparinase and perfused the hearts with albumin (*n* = 5), 6% HES (*n* = 5), or 0.9% normal saline (*n* = 5). The vascular fluid filtration was assessed as epicardial transudate formation. They found the mean levels of transudate formation were dependent on perfusion pressure and significantly lower in the 5% albumin group (2.16 ± 0.42 μl·min^− 1^·cm H_2_O^− 1^) compared to the 0.9% normal saline group (*p* < 0.05, data not shown). Following this study, Jacob et al. [61] reported the protective effect of albumin in an ischemic model of guinea pig hearts. The model treated with albumin had significantly lower mean levels of syndecan-1 (8.8 ± 0.8 g/g dry weight (DW) vs 6.6 ± 0.8 g/g DW, *p* = 0.032) and heparan sulfate (808 ± 176 g/g DW vs 328 ± 61 g/g DW, *p* < 0.001) in the coronary effluent, as compared to non-albumin treated model. We did not identify clinical studies that investigated the utility of albumin administration in preventing glycocalyx degradation in sepsis.

Several preclinical studies found that fresh frozen plasma (FFP) had a protective effect on glycocalyx degradation in non-sepsis models. Kozar et al. [62] demonstrated that FFP inhibited glycocalyx degradation in rats with hemorrhagic shock compared to controls and lactate ringer (LR). They also measured the levels of syndecan-1 mRNA extracted from lung tissue of hemorrhagic shock rats. The syndecan-1 mean mRNA level was significantly reduced in hemorrhagic shock (1.39 ± 0.22) compared to control (3.03 ± 0.22, *p* < 0.02). They also found that the mean level of syndecan-1 mRNA was significantly higher in rats resuscitated with FFP (2.76 ± 0.03) than those resuscitated with LR (0.82 ± 0.03, *p* < 0.001). Others have reported similar findings [63–65]. Conversely, Nelson et al. [66] found no difference in the mean plasma level of syndecan-1 and heparan sulfate in hemorrhagic shock model rats resuscitated with FFP (*n* = 10), albumin (*n* = 9), and Ringer’s acetate (RA; *n* = 9), after adjusting plasma volume difference caused by FFP, albumin, and RA.

Straat et al. investigated the effects of FFP on glycocalyx degradation in critical illness [67]. They compared the median levels of plasma syndecan-1 before and after FFP transfusion (12 ml/kg) in non-bleeding critically ill patients (*n* = 33; 45% of their patients were septic). They found the median level of syndecan-1 after FFP transfusion was significantly lower than before FFP transfusion (565 [IQR 127–1176] pg/mL vs 675 [IQR 132–1690] pg/mL, *p* = 0.01). These data are provocative but clearly further study is warranted.

## Potential therapeutic options for inhibiting glycocalyx degradation in sepsis

Several novel molecules are being investigated as possible glycocalyx-protective therapeutics. As described above, S1P is a sphingolipid that may help improve glycocalyx integrity by inhibiting syndecan-1 shedding. S1P activates S1P_1_ receptor and the activation of S1P_1_ receptor attenuates the activity of MMPs causing syndecan-1 ectodomain shedding [59]. One prior study reported that serum S1P level is decreased in patients with sepsis and septic shock and associated with sepsis severity [68].

Heparin is postulated to protect the glycocalyx from degradation in sepsis by serving as an inhibitor of heparanase, which sheds heparan sulfate from the endothelial glycocalyx. A preclinical study reported that thinning of glycocalyx layer in lung microvessels is due to heparan sulfate degradation induced by TNF-α-dependent heparanase activation; this was attenuated by heparin treatment in a LPS murine model [18]. Since the activation of heparanase can increase the level of MMP expression, heparin also may attenuate the increase of MMP expression levels by inhibiting heparanase activity [27]. Sulodexide (SDX), a highly purified extraction product from porcine intestinal mucosa, has been similarly reported to inhibit heparanase activity [69]. One preclinical study by Song et al. [70] reported that SDX treatment attenuated shedding of heparan sulfate and syndecan-4 in a septic mouse model.

Fibroblast growth factor (FGF) is a mediator of the physiological repair of glycocalyx. It is rapidly activated by circulating heparan sulfate fragments generated by degradation of glycocalyx and binds to FGF receptor, which conducts a signal for activating glycocalyx repairing molecules such as exostosin-1, an enzyme responsible for heparan sulfate synthesis. In sepsis, however, this repair process is significantly delayed because the signaling from activated FGF receptor is inhibited [71]. Enhancing this glycocalyx-repairing signal attenuated by sepsis is a potential therapeutic approach to reconstitute the glycocalyx layer and improve its function [72].

## Conclusions

Glycocalyx degradation is gaining recognition as an important aspect of sepsis pathophysiology. Although the mechanisms of degradation are not fully elucidated, the increased plasma and urine levels of glycocalyx components may serve as diagnostic and prognostic biomarkers in sepsis. Some studies have investigated components that protect the glycocalyx from degradation, while others investigate the possibility of repair of a degraded glycocalyx. The relationship between the degradation and fluid therapy could yield other new insights into the prevention of the degradation. Finally, given the emerging role of the glycocalyx as a central part of sepsis pathophysiology, further studies are needed to establish therapeutic strategies to treat glycocalyx degradation in sepsis.

## Abbreviations

AKI: Acute kidney injury
Ang-1: Angiopoietin 1
Ang-2: Angiopoietin 2
ANP: Atrial natriuretic peptide
APACHE: Acute Physiology and Chronic Health Evaluation
AUC: Area under the curve
DW: Dry weight
FFP: Fresh frozen plasma
FGF: Fibroblast growth factor
GAG: Glycosaminoglycans
HES: Hydroxyethyl starch
ICU: Intensive care unit
IL-10: Interleukin-10
IL-1β: Interleukin-1beta
IL-6: Interleukin-6
IQR: Interquartile range
LPS: Lipopolysaccharide
LR: Lactate ringer
MMPs: Metalloproteinases
mRNA: Messenger ribonucleic acid
OPS: Orthogonal phase spectrometry
PBR: Perfusion boundary region
PG: Proteoglycans
RA: Ringer acetate
ROC: Receiver operating characteristic
ROS: Reactive oxygen species
S1P: Sphingosine-1-phosphate
SDF: Sidestream dark field
SDX: Sulodexide
SIRS: Systemic inflammatory response syndrome
SOFA: Sequential organ failure assessment
TNF-α: Tumor necrosis factor alpha
